# Another view of sequential sampling in the birth process with immigration

**DOI:** 10.1007/s00285-023-02041-0

**Published:** 2024-02-08

**Authors:** Poly H. da Silva, Arash Jamshidpey, Simon Tavaré

**Affiliations:** 1 Irving Institute for Cancer Dynamics, Columbia University, 1190 Amsterdam Avenue, New York, NY 10027 USA; 2 Department of Statistics, Columbia University, 1255 Amsterdam Avenue, New York, NY 10027 USA; 3 Department of Mathematics, Columbia University, 2990 Broadway, New York, NY 10027 USA

**Keywords:** Counts-of-counts data, Yule process with immigration, Poisson marking theorem, Chinese Restaurant Process, Ewens Sampling Formula, Embedding, 60C05, 60J25, 60J80, 92D10, 92D25, 92D40

## Abstract

We explore properties of the family sizes arising in a linear birth process with immigration (BI). In particular, we study the correlation of the number of families observed during consecutive disjoint intervals of time. Letting *S*(*a*, *b*) be the number of families observed in (*a*, *b*), we study the expected sample variance and its asymptotics for *p* consecutive sequential samples $S_p =(S(t_0,t_1),\dots , S(t_{p-1},t_p))$, for $0=t_0<t_1<\dots <t_p$. By conditioning on the sizes of the samples, we provide a connection between $S_p$ and *p* sequential samples of sizes $n_1,n_2,\dots ,n_p$, drawn from a single run of a Chinese Restaurant Process. Properties of the latter were studied in da Silva et al. (Bernoulli 29:1166–1194, 2023. 10.3150/22-BEJ1494). We show how the continuous-time framework helps to make asymptotic calculations easier than its discrete-time counterpart. As an application, for a specific choice of $t_1,t_2,\dots , t_p$, where the lengths of intervals are logarithmically equal, we revisit Fisher’s 1943 multi-sampling problem and give another explanation of what Fisher’s model could have meant in the world of sequential samples drawn from a BI process.

## Introduction

We consider continuous-time models for the evolution of a population of individuals in which each individual belongs to a family. We assume individuals within a family are indistinguishable, so that at time $t >0$ the structure of the population may be recorded by the numbers of families $C_j(t)$ having *j* members at time t, for $j=1,2,\ldots$. There are many classical examples of stochastic processes of this type, including work of Fisher (1943), Kendall (1948, 1949), Karlin and McGregor (1967) and Tavaré (1987). A common feature of these models is their underlying stochastic structure, determined by (a) a stochastic model for the formation of new families, and (b) a stochastic model that describes the evolution of a given family, each assumed to evolve independently. In Karlin and McGregor (1967), examples for (a) include Poisson processes and renewal processes, while for (b) the model can be an integer-valued Markov process.

We have chosen to describe the structure of the model in terms of *individuals* and *families*, but could have just as well used, for example, *specimens* and *species* in an ecological setting, or *genes* and *alleles* in a genetics setting. Another example is provided by the arrival of items at a collection center, the items being classified as a new type or an existing type as they arrive. Other examples will occur to the reader.

In the case where the new families arise at the points of a non-homogeneous Poisson process $\{\mathcal {I}(t), t \ge 0\}$ of rate $\theta (t)$, and each resultant family evolves according to a homogeneous Markov process $\{B(t), t \ge 0\}$ with transition function

$$\begin{aligned} p_{jk}(t) := \mathbb {P}(B(t) = k \vert B(0) = j), j,k = 0,1,2,\ldots , \end{aligned}$$

Karlin and McGregor (1967) showed that for each fixed $t > 0$, the counts $C_j(t)$ are independent Poisson random variables, with means

1 $$\begin{aligned} \mathbb {E}C_j(t) = \int _0^t \, \theta (u) p_{1j} (t-u) du, \end{aligned}$$

a result that is a consequence of the Poisson nature of $\mathcal {I}(t)$. A number of other statistics are often studied, including the number *Z*(*t*) of individuals alive at time *t*,

2 $$\begin{aligned} Z(t) = \sum _{j = 1}^\infty j C_j(t),\quad \mathbb {E}Z(t) = \int _0^t \theta (u) \mathbb {E}B(t-u) du, \end{aligned}$$

and the number *F*(*t*) of families alive at time *t*,

3 $$\begin{aligned} F(t) = \sum _{j=1}^\infty C_j(t), \quad \mathbb {E}F(t) = \int _0^t \theta (u) (1 - p_{10}(t-u)) du. \end{aligned}$$

We note that the process $\{F(t), t \ge 0\}$ is not Markovian, making a detailed analysis of its behavior more complicated. We will return to this later in the paper.

In the case of a homogeneous Poisson input process, the process $C(t) := (C_1(t), C_2(t),\ldots ), t \ge 0)\}$ is a Markov process, and its behavior may be described as follows: transitions are made from $C(t) = c = (c_1, c_2, \ldots )$ to

4 $$\begin{aligned} c + \delta (l,j)&\text { at rate }&c_l q_{lj}, l \ne j \\ c + (1,0,0,\ldots )&\text { at rate }&\theta \nonumber \end{aligned}$$

where $\delta (l,j) = (0,\ldots ,0,-1,0,\ldots ,0,1,0,0,\ldots )$, the -1 is in position *l*, and the 1 in position *j*. The matrix $(q_{ij}, i,j \ge 0)$ is the infinitessimal generator of *B*. The process starts from the state $C(0) = (0,0,0,\ldots )$.

We will focus on the case where new families arise according to a homogeneous Poisson process of rate $\theta$, and families grow according to a pure birth (Yule) process, $B(\cdot )$, with per capita birth rate $\lambda = 1$. (This results in no loss of generality, since for arbitrary $\lambda$ one replaces *t* by $\lambda t$, and $\theta$ by $\theta /\lambda$.) For this example, the so-called birth process with immigration (BI), we have transition rates given by (4), where the generator matrix is

$$\begin{aligned} q_{lj} =\left\{ \begin{array}{cl} l, &{}\text { if } j = l+1;\\ - l, &{}\text { if }j=l; \\ 0 &{}\text { otherwise}; \end{array}\right. \end{aligned}$$

see Kendall (1975, section 4) and Tavaré (1987) for example.

### The jump chain of the BI process

The jump chain $\widetilde{C}_n, n = 0,1,2,\ldots$ of the BI process $C(t), t \ge 0$ is of particular interest to us in the sequel, and we record some basic properties here. It is a discrete-time Markov chain indexed by the number $n = \sum _{j=1}^\infty j c_j \ge 0$ of individuals alive just before the $(n+1)$-st jump time, where as before $c_j$ is the number of families of size *j* at the jump time. The transition probabilities of $\widetilde{C}_n$ are given by

5 $$\begin{aligned} c \rightarrow c + \delta (l,l+1)&\text {with probability }&\frac{ l c_l }{n+\theta }, l = 1,2,\ldots \nonumber \\ c \rightarrow c + (1,0,0,\ldots )&\text {with probability }&\frac{ \theta }{n+\theta }. \end{aligned}$$

The evolution of $\widetilde{C}_n$ may be described intuitively as follows. Think of samples arriving at a collection site one at a time, a first sample, a second, and so on. The first sample to arrive is necessarily of a new type (or family), the second is either the same type (family) as the first with probability $1/(\theta +1)$, or is a new type with probability $\theta /(\theta +1)$. If there are $c_j$ types of size *j* after the *n*th sample has arrived, then the $(n+1)$-st sample is a novel type with probability $\theta /(\theta +n)$, or is a member of an existing type with probability proportional to the number of samples of that type. This process is often referred to as the Chinese Restaurant Process (Aldous 1985), abbreviated CRP in what follows.

It is well known (cf. Tavaré 1987) that given *n* samples have been collected, the joint distribution of the number $\widetilde{C}_j(n)$ of types represented *j* times is given by the Ewens Sampling Formula (Ewens 1972; hereafter, ESF):6for $n\in \mathbb {N}$, where $\theta _{(n)}=\theta (\theta +1)\cdots (\theta +n-1)$ and $\theta _{(0)}=1$.

The original setting for (6) is from population genetics: Ewens (1972) established that the allelic partition of a sample of *n* haploid individuals taken from a well-mixed selectively neutral population of large, constant size *N* and undergoing mutation according to the infinitely-many-alleles (IMA) model of Kimura and Crow (1964) with mutation rate *u* is given approximately by (6). Here, $\theta = 2 N u$ is the compound mutation parameter. For many other settings in which (6) arises, see Arratia et al. (2003).

### Multiple sequential samples in the BI process

The aim of this paper is to investigate the correlation of the number of families observed in some consecutive non-overlapping time intervals of the BI process. Letting *S*(*a*, *b*) be the count of families observed in the interval (*a*, *b*), we focus on computing the expected sample variance and its asymptotic behavior for *p* consecutive sequential samples $S(t_0,t_1),\dots , S(t_{p-1},t_p)$, where $0=t_0<t_1<\dots <t_p$. Our analysis concentrates on two types of sequential time intervals: those of equal size and those of logarithmically equal size, where the latter means $t_0=0$ and $e^{t_i}-e^{t_{i-1}}=\gamma >0$ for $i=1,\ldots ,p$. The Poisson nature of the BI model allows us to derive explicit results concerning the behavior of the number of families arising in *p* sequential time intervals. The analysis relies on simple Marked Poisson Process arguments, for which various marking probabilities may be calculated explicitly. To be more precise, the points of a Poisson process $\xi$ on $\mathbb {R}_+$ with intensity $\theta (x) dx$ can be marked in random by elements of a countable set of marks $\mathcal {M}$ where the probability that each point *x* of $\xi$ is marked by $m\in \mathcal {M}$ is given by $\chi _m(x)$. If the points of $\xi$ are marked independently, then for any $m\in \mathcal {M}$ the points of $\xi$ marked by *m* can be considered as another Poisson process, namely a marked Poisson process, with intensity $\theta (x)\chi _m(x)dx$. This is sometimes called the Marking Theorem (cf. Kallenberg 2002, Theorem 12.10; Kingman 1993, Section 5.2). We apply the Marking Theorem for the point process representing the arriving times of new families in the BI process. For instance, in the beginning of Sect. 3 we study the number of families marked if they are observed in a given time interval or in Lemma 2 we use the Marking Theorem to investigate the number of families marked if they arrived in (*a*, *b*) while they have exactly *j* members at time $t>b$.

da Silva et al. (2023) studied the properties of *p* sequential samples of size $n_i$, $i=1,\dots ,p$, drawn from a single run of a CRP. Their work was motivated by an interpretation of a much older problem of Fisher (1943), who was concerned with finding the expected sample variance of (in our phraseology) $S_1$ and $S_2$, namely $\mathbb {E}V_2 := \mathbb {E}(S_1 - S_2)^2/2$, when the two sample sizes are equal, to *n* say. Fisher surmised that when *n* is large, $\mathbb {E}V_2 \sim \theta \log 2$. For other studies on the correlation of samples of the CRP and the history of Fisher’s problem see also Kendall (1948, 1949), Ewens et al. (2007), Barbour and Tavaré (2010), Lijoi et al. (2008), Sibuya (2014) and McCullagh (2023a, 2023b). As an application of our results, the present paper analyzes Fisher’s problem in a more general setting for a BI process, and provides another view of Fisher’s calculations from a continuous-time perspective. Moreover, we explore the relation between the discrete-time and the continuous-time models through an extended version of an embedding of the CRP into the BI process (cf. Sect. 6).

### Outline of the results

The paper is organized as follows. Section 2 reviews some basic tools for the BI model needed to develop the main results. In Sect. 3, we make use of the Marking Theorem to show the number of families observed in one given interval of time is a Poisson process. We compute the expected value of this Poisson process (Corollary 1), and obtain an exact formula for the covariance of numbers of observed families in two disjoint intervals (Corollary 2). To this end, we show for such two intervals, the number of families observed in both intervals and the number of families observed only in one of them are independent Poisson processes. We obtain the expected values of these Poisson processes in Theorem 1. Section 4 is devoted to sampling in multiple consecutive non-overlapping intervals of time. We make use of the results in Sect. 3 to compute the expected sample variance of the numbers of families observed in such intervals. We apply this, in Sect. 4.1, for specific time intervals whose lengths are logarithmically equal, that is, we consider intervals $[t_0,t_1),[t_1,t_t),\ldots ,[t_{p-1},t_p)$ where $t_0=0$ and $e^{t_i}-e^{t_{i-1}}$ is constant for $i=1,\ldots ,p$.

Section 4.2 studies Fisher’s multiple sampling problem (Fisher 1943) for the BI processes. For a given sequence of $$n_1,n_2,\ldots ,n_p\in \mathbb {Z}_+$$, we first find $$t_0=0<t_1<\cdots <t_p$$ such that the expected number of new birth events in the interval $$[t_{i-1},t_i)$$ be $$n_i$$. This ends up with the intervals of logarithmic lengths $$\log ((\theta +l_i)/(\theta +l_{i-1}))$$, where $$l_i=\sum _{j=1}^in_i$$. Letting $$\widetilde{S}_i$$ be the number of families observed in the *i*-th interval, Theorem 2 provides the expected value of the Poisson random variables $$\widetilde{S}_1,\ldots ,\widetilde{S}_p$$, and computes their expected sample variance. Letting $$n_i=q_in$$ and letting $$n\rightarrow \infty $$, we study the asymptotic behavior of the expected sample variance obtained in Sect. 4.2. We compare this with the results obtained for Fisher’s problem for CRP.

In Sect. 4.4, we compare and analyze the exact and simulated expected sample variances of the numbers of observed families for two different types of consecutive time intervals. We investigate the effects of $\lambda$ and $\theta$ on the expected value of the sample variance. Conditional on having at least one family up to time $t>0$, Sect. 5 discusses the waiting time for a typical random family alive at *t* to have its first birth after time *t*. Section 6 provides an extended version of an embedding of the CRP into the BI, and explores the connections between multi-sampling theories for the discrete-time and continuous-time processes.

## The Yule process with immigration

We record some well-known results for the Yule process with immigration (cf. Tavaré (1987)) at constant rate $\theta$ and birth rate 1. The distribution of the number of members of a typical family that has grown for time *t* from a single individual is geometric:

7 $$\begin{aligned} p_j(t) := \mathbb {P}(B(t) = j \vert B(0) = 1) = e^{-t} (1-e^{-t})^{j-1}, j=1,2,\ldots \end{aligned}$$

As above, we denote the transition probability $\mathbb {P}(B(t) = k \vert B(0) = j)$ by $p_{jk}(t)$, and set $p_j(t) = p_{1j}(t)$.

The population size at time *t* is denoted by *Z*(*t*), and the number of families of size *i* at time *t* is denoted by $C_i(t), i = 1, 2, \ldots$. As noted earlier, for each $t > 0$, the $C_i(t)$ are independent Poisson random variables with means given by

8 $$\begin{aligned} \mathbb {E}C_i(t) = \theta (1-e^{-t})^i / i, i=1,2,\ldots , \end{aligned}$$

as may be verified from (1) and (7). It follows that $Z(t) = \sum _{i = 1}^\infty i C_i(t)$ has a negative binomial distribution (Kendall 1948) with

9 $$\begin{aligned} \mathbb {P}(Z(t) = n) = \left( {\begin{array}{c}\theta +n-1\\ n\end{array}}\right) e^{-\theta t} (1 - e^{-t})^n, n=0,1,2 \ldots , \end{aligned}$$

where

$$\begin{aligned} \left( {\begin{array}{c}\theta +n-1\\ n\end{array}}\right) :=\frac{\Gamma (\theta +n)}{n!\Gamma (\theta )}=\frac{\theta _{(n)}}{n!}. \end{aligned}$$

We make frequent use of the probability generating function (pgf) of *B*(*t*) which is given by

10 $$\begin{aligned} \phi (t;s) := \mathbb {E}s^{B(t)} = \sum _{j \ge 1} p_j(t) s^j = \frac{e^{-t} s}{1 - (1 - e^{-t})s}, \end{aligned}$$

for $$0 \le s \le 1$$.

## Families observable in a given period of time

If we were to watch the growth of a given family through time, there will be some intervals in which no new births are observed. In such an interval, all the members of the family were old, that is, were born before the start of the interval; otherwise, the family is composed of old members and new ones. It is the interplay between these two types that we uncover.

We say a family is *observable* in (*a*, *b*), for $0\le a<b$, if it has at least one birth in (*a*, *b*). Let *S*(*a*, *b*) count the families observable in (*a*, *b*). Of interest is the distribution of *S*(*a*, *b*) and the joint distribution of *S*(*a*, *b*) and *S*(*c*, *d*) for $0\le a<b\le c<d$. In particular, we compute $\textrm{Cov}(S(a,b),S(c,d))$. To this end, for $J\subseteq \mathbb {R}_+$ let $X(J)\sim Poisson(\theta \mid \!\! J\!\!\mid )$ be the number of families initiated in *J*. In particular, for $J=(a,b)$, we have $X(J)=\mathcal {I}(b)-\mathcal {I}(a)$.

Now, letting $I_1=(a,b), I_2=(c,d)$, we notice that

$$\begin{aligned} S(I_1)= & {} S(a,b)=K(I_1,I_2)+T(I_1\setminus I_2),\\ S(I_2)= & {} S(c,d)=K(I_1,I_2)+T(I_2\setminus I_1), \end{aligned}$$

where $K(I_1,I_2)$ counts families observable in both $I_1$ and $I_2$, and $T(I_i\setminus I_j)$ counts families observable in $I_i$ but not in $I_j$, for $\{i\ne j\}=\{1,2\}$. In fact, $K(I_1,I_2)$ counts those points of the Poisson process *X* on (0, *d*), which are marked if observed in both $I_1$ and $I_2$. Similar marking applies to $T(I_1\setminus I_2)$ and $T(I_2\setminus I_1)$. From the Marking Theorem, $K(I_1,I_2)$, $T(I_1\setminus I_2)$ and $T(I_2\setminus I_1)$ are independent Poisson random variables. To obtain their expected values, for $J=(a_0,b_0)$ and $I_i=(a_i,b_i)$ such that $b_{i-1}\le a_i$ for $i=1,\dots ,k$, we define random variables $V_J(I_1,\dots ,I_k)=V_{a_0,b_0}(I_1,\dots ,I_k)$ to count the number of families initiated in *J* and not observable in $I_1,\dots ,I_k$. To simplify the calculations in the proof of the following lemma which will be applied later, we denote

11 $$\begin{aligned} \Phi (a,b,c;s) := \int _a^b \,\phi (c-u;s) du = \log \left( \frac{1 - (1 - e^{-(c-b)}) s}{ 1 - (1 - e^{-(c-a)}) s}\right) . \end{aligned}$$

### Lemma 1

For $0\le a<b\le c<d$, let $J=(a,b)$ and $I=(c,d)$. Then $V_J(I)$ is a Poisson r.v. with mean

$$\begin{aligned} \mathbb {E}V_J(I) = \theta \log \left( \frac{e^d - e^c + e^b}{e^d - e^c + e^a}\right) \end{aligned}$$

### Proof

For $r\in \mathbb {N}$, let $X_{J,r}(I)$ count all those families initiated in *J*, with exactly *r* members at *c*, which are not observable in *I*. The probability that a family initiated at $x\in J$, is not observable in *I*, while it has exactly *r* members at *c* is given by

$$\begin{aligned} p_{r}(c-x)e^{-r (d-c)}. \end{aligned}$$

Hence, from the Marking Theorem, for $r\in \mathbb {N}$, $V_{J,r}(I)$ are independent Poisson r.v.s with expected value

$$\begin{aligned} \mathbb {E}V_{J,r}(I) = \theta \int _J p_{r}(c-x)e^{-r(d-c)}dx. \end{aligned}$$

As a consequence, $V_J(I)$ is a Poisson r.v. with parameter

$$\begin{aligned} \mathbb {E}V_J(I)= & {} \sum _{r=1}^\infty \mathbb {E}V_{J,r}(I) = \theta \int _a^b \phi (c-x;e^{-(d-c)}) dx \\= & {} \theta \Phi (a,b,c;e^{-(d-c)}) = \theta \log \left( \frac{e^d - e^c + e^b}{e^d - e^c + e^a}\right) , \end{aligned}$$

using (11) and simplifying. $\square$

We highlight one special case of this result for the case $J = (0,a), I = (a,b)$. Lemma 1 gives

$$\begin{aligned} \mathbb {E}V_J(I) = \theta b - \theta \log (e^b - e^a + 1). \end{aligned}$$

We can establish the following lemma in a similar way.

### Lemma 2

For $0\le a<b\le c<d$, let $J=(0,a)$, $I_1 = (a,b)$ and $I_2=(c,d)$. Then $V_J(I_1,I_2)$ is a Poisson r.v. with expected value

$$\begin{aligned} \mathbb {E}V_J(I_1,I_2) = \theta \log \left( \frac{e^d - e^c + e^b}{e^d - e^c + e^b - e^a +1}\right) \end{aligned}$$

### Proof

The probability that a family arrives in *J* and is not observable in $I_1$ and $I_2$, while it has *r* and *s* members at *a* and *c*, respectively, $1\le r\le s$, is given by

$$\begin{aligned} p_r(a-x)e^{- r(b-a)}p_{rs}(c-b)e^{- s(d-c)}. \end{aligned}$$

Hence, from the Marking Theorem, for $b<c$, $V_J(I_1,I_2)$ is a Poisson r.v. with

$$\begin{aligned} \mathbb {E}V_J(I_1,I_2)= & {} \sum _{s\ge 1}\sum _{r\le s}\theta \int _0^a p_{r}(a-x)e^{-r(b-a)} p_{rs}(c-b)e^{-s(d-c)}dx\\= & {} \theta \int _0^a \sum _{r\ge 1} p_{r}(a - x) e^{-r(b-a)} \sum _{s\ge r} p_{rs}(c-b) e^{-s(d-c)} dx\\= & {} \theta \int _0^a \sum _{r\ge 1} p_{r}(a - x) e^{-r(b-a)} [\phi (c-b;e^{-(d-c)})]^r dx \\= & {} \theta \Phi (0,a,a;e^{-(b-a)}\phi (c-b;e^{-(d-c)})), \end{aligned}$$

the second to last line coming from the fact that families evolve independently. Using (11) once more, and simplifying, we get

$$\begin{aligned} \mathbb {E}V_J(I_1,I_2) = \theta \log \left( \frac{e^d - e^c + e^b}{e^d - e^c + e^b - e^a +1}\right) , \end{aligned}$$

as required. The case $b = c$ reduces to $\mathbb {E}V_J(I)$ for $J = (0,a), I = I_1 \cup I_2 = (a,d),$ given in Lemma 1. $\square$

To compute the expected values of $K(I,J), T(I\setminus J)$ and $T(J\setminus I)$, we also need to define the random variables $U_J(I_1,\dots , I_k)$ to count the families initiated in *J* and observable in $I_1,\dots ,I_k$.

### Lemma 3

For $0\le a<b\le c<d$, let $J=(a,b)$ and $I=(c,d)$. Then $U_J(I)$ is a Poisson r.v. with parameter

$$\begin{aligned} \mathbb {E}U_J(I) = \theta \log \left( \frac{e^b(e^d - e^c + e^a)}{e^a(e^d - e^c + e^b)}\right) \end{aligned}$$

### Proof

The Marking Theorem shows that $U_J(I)$ and $V_J(I)$ are independent Poisson r.v.s. The result follows since

$$\begin{aligned} X(J) = U_J(I) + V_J(I)\sim Poisson(\theta (b-a)).\end{aligned}$$

$$\square $$

### Theorem 1

For $0\le a<b\le c<d$, let $I_1=(a,b)$ and $I_2=(c,d)$. Then $K(I_1,I_2)$, $T(I_1\setminus I_2)$ and $T(I_2\setminus I_1)$ are independent Poisson random variables with means

$$\begin{aligned} \begin{aligned}&\mathbb {E}K(I_1,I_2) = \theta \log \left( \frac{(e^b - e^a + 1)(e^d -e^c + 1)}{e^d - e^c + e^b -e^a + 1}\right) , \\&\mathbb {E}T(I_1\setminus I_2) = \theta \log \left( \frac{e^d - e^c + e^b -e^a + 1}{e^d -e^c + 1} \right) ,\\&\mathbb {E}T(I_2\setminus I_1)=\theta \log \left( \frac{e^d - e^c + e^b -e^a + 1}{e^b -e^a + 1} \right) .\\ \end{aligned} \end{aligned}$$

### Proof

Let $$J=(0,a), J'=(b,c)$$, $$K(I_1,I_2)=U_{I_1}(I_2)+U_J(I_1,I_2)$$, where

$$\begin{aligned} U_J(I_1,I_2)=X(J)-V_J(I_1)-V_J(I_2)+V_J(I_1,I_2). \end{aligned}$$

From Lemmas 1 and 2, we have

$$\begin{aligned} \mathbb {E}U_J(I_1,I_2)= & {} \theta (a-b) + \theta \log (e^b - e^a + 1) \\{} & {} - \theta \log (e^d - e^c + e^a) + \theta \log (e^d - e^c + 1)\\{} & {} + \theta \log (e^d - e^c + e^b) - \theta \log (e^d - e^c + e^b -e^a + 1). \end{aligned}$$

$\mathbb {E}K(I_1,I_2)$ follows after using Lemma 3 and simplifying.

On the other hand,

$$\begin{aligned} T(I_1\setminus I_2)= & {} V_{I_1}(I_2)+V_J(I_2)-V_J(I_1,I_2),\\ T(I_2\setminus I_1)= & {} V_J(I_1)-V_J(I_1,I_2)+U_{J'}(I_2)+X(I_2), \end{aligned}$$

which, applying Lemmas 1–3 and simplifying, gives the result. $\square$

### Corollary 1

For any $0\le a<b$, *S*(*a*, *b*) is a Poisson random variable with mean

$$\begin{aligned} \mathbb {E}S(a,b)= \theta \log (e^b - e^a +1). \end{aligned}$$

### Proof

Letting $I=(a,b)$, the corollary follows from Lemma 3 and the fact that $S(a,b)=U_{(0,a)}(I)+X(I)$. Note that for an interval (0, *b*), *S*(0, *b*) has a Poisson distribution with mean $\theta \log (e^b -e^0 +1) = \theta \log (e^b) = \theta b,$ as it must. $\square$

### Corollary 2

For $0\le a< b\le c<d$, we have

$$\begin{aligned} \textrm{Cov}(S(a,b),S(c,d))= \theta \log \left( \frac{(e^b -e^a + 1) (e^d - e^c + 1)}{e^d -e^c + e^b -e^a + 1}\right) . \end{aligned}$$

### Proof

It is clear from Theorem 1 that

$$\begin{aligned}\textrm{Cov}(S(a,b),S(c,d))=\textrm{Var} K((a,b),(c,d)). \end{aligned}$$

$$\square $$

## Sampling in multiple intervals

We consider sampling from *p* intervals determined by the points $t_0=0<t_1<\dots <t_p$. Let $\delta _i=t_i-t_{i-1}$ for $1\le i\le p$. For $i=1,\dots ,p$, let $S_i := S(t_{i-1},t_i)$ be the number of families observable in the time interval $(t_{i-1},t_i)$. The sample variance of the $S_i$ is given by

$$\begin{aligned} V_p := V_p(t_1,\ldots ,t_p) = \frac{1}{p(p-1)} \sum _{1\le i<j\le p} (S_i - S_j)^2. \end{aligned}$$

We can exploit the previous results to compute $\mathbb {E}V_p$. Since

$$\begin{aligned} \mathbb {E}(S_i - S_j)^2 = \textrm{Var} S_i + \textrm{Var} S_j - 2 \textrm{Cov}(S_i,S_j) + (\mathbb {E}S_i - \mathbb {E}S_j)^2, \end{aligned}$$

we see from Corollaries 1 and 2 that

12 $$\begin{aligned} \mathbb {E}V_p(t_1,\ldots ,t_p)= & {} \frac{1}{p(p-1)} \, \sum _{i<j} \left\{ \theta \log \left( \frac{(e^{t_j} -e^{t_{j-1}} + e^{t_i} - e^{t_{i-1}} + 1)^2}{(e^{t_j} -e^{t_{j-1}} + 1)(e^{t_i} - e^{t_{i-1}} + 1)}\right) \right. \nonumber \\{} & {} \quad + \left. \theta ^2 \log ^2\left( \frac{e^{t_i} - e^{t_{i-1}} + 1}{e^{t_j} -e^{t_{j-1}} + 1} \right) \right\} . \end{aligned}$$

If the time intervals are of equal length, say $t_i = i \tau /p, i=0,1,\ldots ,p$ then (12) reduces to

13 $$\begin{aligned} \mathbb {E}V_p(\tau )= & {} \frac{\theta }{p(p-1)} \, \sum _{i<j} \log \left( \frac{(\gamma ^j -\gamma ^{j-1} + \gamma ^i - \gamma ^{i-1} + 1)^2}{(\gamma ^j - \gamma ^{j-1} + 1)(\gamma ^i - \gamma ^{i-1} + 1)}\right) \nonumber \\{} & {} + \frac{\theta ^2}{p(p-1)} \sum _{i<j} \log ^2\left( \frac{\gamma ^i - \gamma ^{i-1} + 1}{\gamma ^j - \gamma ^{j-1} + 1}\right) , \end{aligned}$$

where $$\gamma = e^{\tau /p}.$$

### Logarithmically equal interval lengths

There is one special case that results in the $S_i$ being identically distributed, namely the setting in which $e^{t_i} = i \gamma +1$ for $i=0,1,\cdots , p$ and for some $\gamma > 0$; from Corollary 1, the $S_i$ then have Poisson distributions with

$$\begin{aligned} \mathbb {E}S_i = \theta \log (\gamma +1), i=1,2,\ldots ,p, \end{aligned}$$

and, from Corollary 2, covariances given by

$$\begin{aligned} \text {Cov}(S_i,S_j) = \theta \log \left( \frac{(\gamma +1)^2}{2 \gamma +1}\right) , i \ne j. \end{aligned}$$

As a consequence, the correlation between $S_i$ and $S_j$ is given by

$$\begin{aligned} \rho := \rho (S_i,S_j) = 2 - \log (2\gamma +1)/\log (\gamma +1), i \ne j, \end{aligned}$$

and, from (12),

14 $$\begin{aligned} \mathbb {E}V_p = \theta \log \left( \frac{2 \gamma + 1}{\gamma +1}\right) . \end{aligned}$$

Given the counts $S_i, i = 1,\ldots ,p$, we let $\bar{S}$ be their mean, so that $\mathbb {E}\bar{S} = \theta \log (1+\gamma )$. This suggests a Watterson-type estimator of $\theta$ given by

15 $$\begin{aligned} \theta _S = \bar{S}/\log (1+\gamma ) \end{aligned}$$

(Watterson 1975). The estimator is unbiased, but as $p \rightarrow \infty$,

$$\begin{aligned} \textrm{Var}\, \theta _S = \textrm{Var}\, \bar{S}/\log ^2(1+\gamma ) = \frac{\theta }{p \log (\gamma +1)} (1 + (p-1)\rho ) \rightarrow \frac{\theta \rho }{\log (\gamma +1)}. \end{aligned}$$

### Fisher’s problem revisited

Here we revisit Fisher’s sampling problem Fisher (1943). Several approaches have appeared in the literature, and we point the reader to da Silva et al. (2023) for an overview. Here we provide an alternative setting, via the Yule process with immigration, which leads to rather transparent connections between the different views.

The setting described in da Silva et al. (2023) occurs in discrete time, where successive samples of individuals of sizes $n_1, n_2, \ldots , n_p$ are taken, and the observations are $S^*_i, i=1, 2, \ldots ,p$, $S^*_i$ denoting the number of distinct types observed in the *i*th sample. The generative model is that of the CRP, a sequential model in which the distribution of the counts of family sizes follows the Ewens Sampling Formula (Ewens 1972). It is of interest to compare the two settings.

To do this, recall from (9) that *Z*(*t*) has a negative binomial distribution with

16 $$\begin{aligned} \mathbb {E}Z(t) = \theta (e^t - 1). \end{aligned}$$

We could choose the time points $0 = t_0, t_1,\ldots ,t_p$ in such a way that the cumulative number of individuals observed, $l_i = n_1+\cdots +n_i, i = 1,2,\ldots ,p$ and $l_0 = 0$, matches the expectation under the Yule model. To this end, we solve

$$\begin{aligned} \mathbb {E}Z(t_i) = \theta \left( e^{t_i}-1\right) =l_i, \ i=1,\dots ,p. \end{aligned}$$

to get

$$\begin{aligned} \delta _i= t_i - t_{i-1} = \log \left( \frac{\theta +l_i}{\theta +l_{i-1}}\right) , \end{aligned}$$

and

17 $$\begin{aligned} t_i=\log \left( \frac{\theta +l_i}{\theta }\right) , \end{aligned}$$

for $$i=1,\dots ,p$$.

We remark that if $\theta$ were known, and we wish to estimate the time points $t_i$ given the counts $n_i$, then (17) gives the moment estimators of the $t_i$, and this in turn provides the maximum likelihood estimators of the $t_i$.

Henceforth we assume that $t_i=\log (\theta +l_i) - \log \theta$, and define $\widetilde{S}_i = S(t_{i-1},t_i)$, $i=1,\dots ,p$, and

$$\begin{aligned} \widetilde{V}_p= \widetilde{V}_p(n_1,\dots ,n_p):=\frac{1}{p(p-1)}\sum _{i<j}(\widetilde{S}_i-\widetilde{S}_j)^2. \end{aligned}$$

The results of Sect. 3 translate into

#### Theorem 2

For any $i\in \mathbb {N}$, $\widetilde{S}_i$ is a Poisson random variable with mean

$$\begin{aligned} \mathbb {E}\widetilde{S}_i=\theta \log \left( \frac{\theta +n_i}{\theta }\right) . \end{aligned}$$

Furthermore, for any $p,i,j\in \mathbb {N}$, $i\ne j$,

$$\begin{aligned}{} & {} \textrm{Cov}(\widetilde{S}_i,\widetilde{S}_j)=\theta \log \left( \frac{(\theta +n_i)(\theta +n_j)}{\theta (\theta +n_i+n_j)}\right) ,\\{} & {} \mathbb {E}\widetilde{V}_p=\frac{1}{p(p-1)}\sum _{1\le i<j\le p}\left\{ \theta \log \left( \frac{(\theta +n_i+n_j)^2}{(\theta +n_i)(\theta +n_j)}\right) +\theta ^2\log ^2\left( \frac{\theta +n_i}{\theta +n_j}\right) \right\} . \end{aligned}$$

#### Proof

The results follow from substituting $t_i = \log (\theta +l_i) - \log \theta$ in Corollary 1, Corollary 2 and (12), respectively, and simplifying. $\square$

#### Corollary 3

Let $$n_1=n_2=\dots =n_p=n$$. Then

$$\begin{aligned} \mathbb {E}\widetilde{V}_p =\theta \log \left( \frac{2n+\theta }{n+\theta }\right) . \end{aligned}$$

The term on the right appears in Fisher (1943, p. 451, after (5)) for the case $p=2$, so our model provides a unifying approach to Fisher’s question.

### Asymptotic behavior

To see the asymptotic behavior, let $n_i=q_in$, $i=1,\dots , p$, where $q_i \in (0,1)$ satisfy $q_1+\cdots +q_p = 1$. As $n\rightarrow \infty$, we see that

$$\begin{aligned} \textrm{Cov}(\widetilde{S}_i,\widetilde{S}_j)\sim \theta \log \left( \frac{n_in_j}{n_i+n_j}\right) =\theta \log n+\theta \log \frac{q_iq_j}{q_i+q_j}, \end{aligned}$$

and

$$\begin{aligned}{} & {} \mathbb {E}\widetilde{V}_p=\frac{1}{p(p-1)}\sum _{1\le i<j \le p} \left\{ \theta \log \left( \frac{(\theta +n(q_i+q_j))^2}{(\theta +n q_i)(\theta +n q_j)}\right) +\theta ^2\log ^2\left( \frac{\theta +q_i n}{\theta +q_j n}\right) \right\} \\{} & {} \quad \longrightarrow \frac{1}{p(p-1)}\sum _{1\le i<j \le p} \left\{ \theta \log \left( \frac{(q_i+q_j)^2}{q_iq_j}\right) +\theta ^2\log ^2\left( \frac{q_i}{q_j}\right) \right\} . \end{aligned}$$

The righthand formula was derived originally in Barbour and Tavaré (2010) by a different Poisson argument, and as a limit in the discrete case in da Silva et al. (2023).

To compute the rate of convergence, we note that the absolute value of the difference between $\mathbb {E}\widetilde{V}_p$ and the limit is given by

$$\begin{aligned}{} & {} \frac{1}{p(p-1)}\left| \sum _{1\le i<j \le p} \theta \log \left( \frac{\left( 1+\theta /((q_i+q_j)n)\right) ^2}{\left( 1+\theta /(q_in)\right) \left( 1+\theta /(q_jn)\right) }\right) \right. \\{} & {} \quad \left. +\sum _{1\le i<j \le p} \theta ^2\log \left( \frac{1+\theta /(q_in)}{1+\theta /(q_jn)}\right) \left[ 2\log \left( \frac{q_i}{q_j}\right) +\log \left( \frac{1+\theta /(q_in)}{1+\theta /(q_jn)}\right) \right] \right| = O(n^{-1}), \end{aligned}$$

where the order of error is obtained using the Taylor series of $\log (1+c/n)$ for different choices of *c*.

For $$n_1=\dots =n_p$$, as $$n\rightarrow \infty $$,

$$\begin{aligned} \textrm{Cov}(\widetilde{S}_i,\widetilde{S}_j)\sim \theta \log n-\theta \log 2, \end{aligned}$$

and

$$\begin{aligned} \mathbb {E}\widetilde{V}_p\longrightarrow \theta \log 2, \end{aligned}$$

as found by Fisher (1943). Once again the rate of convergence can be computed from the bound

$$\begin{aligned} \left| \theta \log \left( \frac{2n+\theta }{n+\theta }\right) -\theta \log 2\right| =\left| \theta \log \left( 1-\frac{\theta }{2(n+\theta )}\right) \right| = O(n^{-1}). \end{aligned}$$

### Empirical sample variance for two types of intervals

In this section, we allow the birth rate $\lambda$ to be arbitrary, as opposed to setting $\lambda = 1$. We compare the exact and simulated expected values of the sample variance of $S(t_0,t_1),S(t_1,t_2),\ldots , S(t_{p-1},t_p)$, and those of the sample variance of $S(r_0,r_1),S(r_1,r_2),\ldots , S(r_{p-1},r_p)$, for two types of non-overlapping consecutive intervals determined by $0=t_0<t_1<\cdots <t_p=\tau$ and $0=r_0<r_1<\cdots <r_p=\tau$, respectively, for $i=1,\ldots , p$, $t_i ={i \tau }/{p}$ and

$$\begin{aligned} r_i = \frac{1}{\lambda }\log \left( \frac{i (e^{\lambda \tau }-1)}{p}+1\right) . \end{aligned}$$

It is clear that the intervals of the first type are of equal size, while the sizes of the second type of the intervals are logarithmically equal, meaning that $e^{\lambda r_i}-e^{\lambda r_{i-1}}$ is a constant for $i=1,\ldots , p$.

The exact value of $\mathcal {V}:=\mathbb {E}V_p(t_1,\ldots ,t_p)$ can be easily obtained from (13) letting $\gamma =e^{\lambda \tau /p}$ and replacing $\theta$ by $\theta /\lambda$. On the other hand, likewise (12), we can see that

$$\begin{aligned} \mathcal {V}':=\mathbb {E}V_p(r_1,\ldots ,r_p)=\frac{\theta }{\lambda }\log \left( \frac{2\widetilde{\gamma }+1}{\widetilde{\gamma }+1}\right) , \end{aligned}$$

where $$\widetilde{\gamma }=(e^{\lambda \tau }-1)/p$$.

The expected number of individuals in the BI process grows exponentially in time. Consequently, when we consider intervals of equal size, we expect that the number of families observed in the later time intervals is more than the number of families observed in the earlier time intervals. This results in a larger average sample variance. On the other hand, for logarithmically equal interval lengths, we expect the number of observed families to follow an identical distribution, leading to a smaller average sample variance. To illustrate the difference between the sampling from these two types of intervals and also to better understand the interplay between the birth and immigration rates, we simulated a BI process for different values of $\theta$ and $\lambda$. The results are presented in Table 1. It is important to note that the empirical averages of the sample variances presented here are derived from simulations and are subject to statistical errors, while the exact expected values of the sample variances are given by (12).

**Table 1:** Empirical and exact expected values of sample variance of 10 samples of either equal interval lengths or logarithmically equal interval lengths for different values of $\lambda$ and $\theta$

		Equal size		Logarithmically equal size	
$\lambda$	$\theta$	Empirical $\mathbb {E}V_{10}$	Exact $\mathbb {E}V_{10}$	Empirical $\mathbb {E}V_{10}$	Exact $\mathbb {E}V_{10}$
0.1	0.25	1.851	1.892	0.997	1.060
0.1	0.5	5.908	5.400	2.208	2.120
0.1	1	17.286	17.259	4.426	4.239
0.25	0.25	4.051	3.986	0.708	0.684
0.25	0.5	12.910	13.600	1.481	1.367
0.25	1	52.107	49.713	2.651	2.734
0.5	0.25	3.760	4.603	0.261	0.347
0.5	0.5	17.430	16.096	0.725	0.693

The exact expected value of sample variance are obtained from (12). Estimates are based on 50 runs and $\tau =25$

In the case of equal interval lengths, for a fixed $\theta >0$, the expected value of the sample variance increases as $\lambda$ increases. In contrast, in the logarithmic case, an increase in $\lambda$ results in a decrease in the mean of the sample variance. In fact, it is easy to see that as $\lambda$ tends to $\infty$, the expected value of the sample variance converges to 0. In both cases, as $\theta$ increases, the expected value of the sample variance increases with a more substantial effect observed in the case of the equal interval lengths.

The following asymptotic result for $\mathcal {V}$ and $\mathcal {V}'$ can be readily obtained.

#### Proposition 1

For fixed $\theta ,\tau ,p$, as $\lambda \rightarrow \infty$ we have

$$\begin{aligned} \mathcal {V}\rightarrow & {} \frac{\theta \tau (\theta \tau +2)(p+1)}{12p},\\ \mathcal {V}'\rightarrow & {} 0. \end{aligned}$$

#### Proof

As $\lambda \rightarrow \infty$, we have $\widetilde{\gamma }\rightarrow \infty$, hence

$$\begin{aligned} \mathcal {V}'=\frac{\theta }{\lambda }\log \left( \frac{2\widetilde{\gamma }+1}{\widetilde{\gamma }+1}\right) \rightarrow 0. \end{aligned}$$

For the first limit, for $\gamma =\exp (\lambda \tau /p)$, as $\lambda \rightarrow \infty$

$$\begin{aligned}{} & {} \mathcal {V}\sim \frac{1}{p(p-1)}\sum _{1\le i<j\le p} \left\{ \frac{\theta }{\lambda }\log \left( \frac{\gamma ^{2j}}{\gamma ^{i+j}}\right) +\left( \frac{\theta }{\lambda }\right) ^2\log ^2\left( \frac{\gamma ^i}{\gamma ^j}\right) \right\} \\{} & {} \quad \rightarrow \frac{1}{p(p-1)}\left\{ \frac{\theta \tau }{p}\sum _{1\le i<j\le p} (j-i)+\left( \frac{\theta \tau }{p}\right) ^2\sum _{1\le i<j\le p} (j-i)^2\right\} . \end{aligned}$$

Note that the first and the second sums in the limit can be simplified to $p(p^2-1)/6$ and $p^2(p^2-1)/12$, which finishes the proof.

## How long are gaps in arrivals?

In this section we consider the waiting time for a family to have another birth. One setting for this is the following. Conditional on at least one family arriving in (0, *t*), choose one of those families at random. What is the distribution of the length of time for which that family is unobservable after time *t*? Hence, we seek the distribution of the waiting time, $W_t$, for that family to have its first birth after time *t*. We assume $\lambda = 1$ in the sequel.

We begin by showing that $N_t$, the number of members at time *t* of a family randomly chosen in (0, *t*), conditional on having at least one family, is log-series distributed, with parameter $q_t = 1 - e^{-t}$. Since the arrival time in (0, *t*) of a typical family is uniform on (0, *t*), we see that

18 $$\begin{aligned} \mathbb {P}(N_t = j)= & {} \int _0^t \frac{1}{t} \, p_j(t-u) du \nonumber \\= & {} \frac{1}{t} \int _0^t e^{-(t-u)} (1 - e^{-(t-u)})^{j-1} du \nonumber \\= & {} \frac{1}{t} \frac{q_t^j}{j} = \frac{1}{- \log (1 - q_t)} \frac{q_t^j}{j}, \end{aligned}$$

as required.

Since each of the $N_t$ individuals in the family at time *t* behave independently, it follows that, given $N_t = n$, $W_t$ is the minimum of *n* independent unit exponential lifetimes, which is exponential with parameter *n*. Hence, conditional on having at least one family before time *t*, the density $f_t(s)$ of $W_t$ is

19 $$\begin{aligned} f_t(s)= & {} \sum _{n \ge 1} \frac{1}{- \log ( 1 - q_t)} \frac{q_t^ n}{n} \, \cdot ne^{-sn}\\= & {} \sum _{n\ge 1}\frac{(1-e^{- t})^ne^{-sn}}{ t}\nonumber \\= & {} \frac{(1-e^{- t})e^{-s}}{ t (1-e^{-s}(1-e^{- t}))}, \quad s>0. \nonumber \end{aligned}$$

As a result the probability distribution function of $W_t$ can be written as

$$\begin{aligned} \mathcal {F}_t(u)= & {} \mathbb {P}(W_t\le u)=1-\int _u^\infty f_t(s)ds\\= & {} \frac{1}{ t}\log \left( e^{-u}+e^{ t}-e^{-u+ t}\right) ;\quad u\ge 0. \end{aligned}$$

This allows the moments of $W_t$ to be written down immediately:

$$\begin{aligned} \mathbb {E}W_t = \frac{1}{t} \sum _{n \ge 1} \frac{q_t^n}{n^2} = \frac{1}{t} \textrm{Li}_2(q_t),\quad \textrm{Var} W_t = \frac{1}{t} \sum _{n \ge 1} \frac{q_t^n}{n^3} = \frac{1}{t} \textrm{Li}_3(q_t), \end{aligned}$$

where $\textrm{Li}_n(x)$ denotes the polylogarithm function $\textrm{Li}_n(x) = \sum _{k\ge 1} x^k k^{-n}.$

## Conditioning in the Yule process with immigration

da Silva et al. (2023) provide a discrete approach to understand Fisher’s multi-sampling problem. In this paper we tackled Fisher’s problem with a continuous approach. This section discusses the connections between two approaches, focusing primarily on various versions of embedding. The main result is given in Theorem 3 and the discussion after that which explicitly explains the connection between the continuous-time and discrete-time multi-sampling approaches.

To set the scene, recall the Ewens Sampling Formula (ESF) from (6): for $\theta >0$,for $n\in \mathbb {N}$, where $\theta _{(n)}=\theta (\theta +1)\cdots (\theta +n-1)$ and $\theta _{(0)}=1$. The ESF may be realised by conditioning independent Poisson random variables on a finite (Watterson 1974a) or an infinite (Shepp and Lloyd 1966) weighted sum. Hence we discuss a unifying approach that connects both types of conditioning relations through embedding of the CRP into the Yule process with immigration.

To establish the conditioning relations, for $x\in (0,1]$, let $\pi _1(x),\pi _2(x),\dots$ be independent Poisson random variables with $\mathbb {E}\pi _i(x)=\theta x^i/i$, and let $T_n(x)=\sum _{i=1}^n i\pi _i(x)$ and $T_\infty (x)=\sum _{i=1}^\infty i\pi _i(x)$. Extending the result of Shepp and Lloyd (1966), as $T_\infty (x)$ is almost surely finite for $x\in (0,1)$, we have

20 $$\begin{aligned} \mathcal {L}(\pi _1(x),\dots ,\pi _n(x)\mid T_\infty (x)=n)=\mathcal {E}_n, \ \ x\in (0,1). \end{aligned}$$

By conditioning on $T_n(x)$ instead of the infinite sum, Watterson introduced a conditioning relation that holds for $x\in (0,1]$, more precisely

21 $$\begin{aligned} \mathcal {L}(\pi _1(x),\dots ,\pi _n(x)\mid T_n(x)=n)=\mathcal {E}_n, \ \ x\in (0,1]. \end{aligned}$$

Notice from (2) that if we define $\pi _i(x)=C_i(t)$ for $t\in \mathbb {R}_+$ and $x = 1-e^{-t}\in (0,1)$, and let $Z_n(t)=\sum _{i=1}^n i C_i(t)$, by definition we have $T_n(x) = Z_n(t)$ and $T_\infty (x) = Z(t)$. Hence, for $x\in (0,1)$ and $t\in \mathbb {R}_+$, the conditional distribution of counts of family sizes, given $Z(t)=n$ or $Z_n(t)=n$, is the ESF once more. Note that as the $C_i(t)$ are independent Poisson random variables with $\mathbb {E}C_i(t)= \theta (1-e^{-t})^i/i$,

$$\begin{aligned} (C_1(t),C_2(t),\dots )\Rightarrow (C_1(\infty ),C_2(\infty ),\dots ) \end{aligned}$$

where $C_i(\infty )=\pi _i(1)$ has a Poisson distribution with mean $\theta /i$, for $i\in \mathbb {N}$; hence one can derive (21) for $x=1$, by letting $t\rightarrow \infty$.

The conditioning relation provides one way to simulate observations from the ESF: simulate the $C_i(t)$, and accept the realisation if $Z(t) = n$. To make the simulation as efficient as possible, we should choose *t* to make the probability of the conditioning event as large as possible. To do this, we note that the value of *t* may be chosen as a function of *n*, since this choice plays no role in the conditional distribution. Maximizing $\mathbb {P}(Z(t) = n)$ given in (9) gives $t = \log ((n+\theta )/\theta )$ (and so $x = x_n = n/(n+\theta )$). We note that this is the same choice of *t* as provided in (17). We also note that there are far more efficient ways to simulate the ESF; see Arratia et al. (2018) for example.

The conditioning relation (21) is a result of the embedding of the CRP in the Yule process with immigration. Here we discuss embeddings at multiple time points. To this end, let

$$\begin{aligned} \tilde{\mathcal {C}}(n)=(\tilde{C}_1(n),\tilde{C}_2(n),\dots ), \quad n\in \mathbb {N}\end{aligned}$$

denote the counts of family sizes generated by the first *n* arrivals in the CRP with parameter $\theta$, and let

$$\begin{aligned} \mathcal {C}(t)=(C_1(t),C_2(t),\dots ), \quad t\ge 0 \end{aligned}$$

be the family size counts at time *t* of the Yule process with immigration with birth rate 1 and immigration rate $\theta$. To discretize the process $\mathcal {C}=(\mathcal {C}(t), t \ge 0)$, let

$$\begin{aligned} \Psi (n)=(\Psi _1(n),\dots ,\Psi _n(n),0,0,\dots ) \end{aligned}$$

be the family size counts of the first *n* individuals born in the Yule process with immigration, i.e. $\Psi _j(n)$ gives the number of families of size *j*, considering only the first *n* individuals. As a discrete process, $\Psi :=(\Psi (n), n\ge 1)$ records the outcomes of jumps in the Yule process with immigration. This is a slightly different version of the jump Markov chain $J=(J(n), n\ge 1)$ used in Tavaré (1987, Section 3), in which, for each *n*, the families in *J*(*n*) are also sorted in order of their appearance in the population. In other words, for any $n\in \mathbb {N}$, one can easily obtain $\Psi (n)$ from *J*(*n*) by forgetting the order (age) of the families in *J*(*n*) and grouping all families of the same size together. From Theorem 2 in Tavaré (1987), *J* is independent of *Z*, then as a functional of *J*, so is $\Psi$. The independence comes as a result of the fact that at each jump time, one new individual will be added to the existing population, no matter if the new individual arrives as a result of an immigration (new family) or a birth. The connection between $\tilde{\mathcal {C}}=(\tilde{\mathcal {C}}(n), n\in \mathbb {N})$ and $\mathcal {C}=(\mathcal {C}(t)), t \ge 0)$ is given in the next theorem.

### Theorem 3

For any $p\in \mathbb {N}$, $0<t_1<\dots <t_p$, and $0\le l_1\le l_2\le \dots \le l_p$, $l_i\in \mathbb {Z}_+$, and $u_1,u_2,\dots ,u_p\in \mathbb {Z}_+^\mathbb {N}$, we have

22 $$\begin{aligned}{} & {} \mathbb {P}(\mathcal {C}(t_1)=u_1,\dots ,\mathcal {C}(t_p)=u_p\mid Z(t_1)=l_1,\dots ,Z(t_p)=l_p)\nonumber \\{} & {} \quad =\mathbb {P}(\tilde{\mathcal {C}}(l_1)=u_1,\dots ,\tilde{\mathcal {C}}(l_p)=u_p). \end{aligned}$$

### Proof

First note that if $Z(t_1)=l_1,\dots ,Z(t_p)=l_p$, then $\Psi (l_i)=\mathcal {C}(t_i)$, for $i=1,\dots , p$. On the other hand, (3.1) in Tavaré (1987) and the connection between $\Psi$ and *J* shows that $(\Psi (i), i\ge 1)\sim (\tilde{\mathcal {C}}(i), i\ge 1)$ in distribution. Also, from Theorem 2 in Tavaré (1987) *J* and *Z*, and hence $\Psi$ and *Z* are independent processes. Thus we can write

$$\begin{aligned} \frac{\mathbb {P}(\mathcal {C}(t_i)=u_i,Z(t_i)=l_i;i=1,\dots ,p)}{\mathbb {P}(Z(t_i)=l_i;i=1,\dots ,p)}= & {} \frac{\mathbb {P}(\Psi (l_i)=u_i,Z(t_i)=l_i;i=1,\dots ,p)}{\mathbb {P}(Z(t_i)=l_i;i=1,\dots ,p)}\\= & {} \mathbb {P}(\Psi (l_1)=u_1,\dots ,\Psi (l_p)=u_p)\\= & {} \mathbb {P}(\tilde{\mathcal {C}}(l_1)=u_1,\dots ,\tilde{\mathcal {C}}(l_p)=u_p). \end{aligned}$$

$$\square $$

To better connect the sequential multi-sampling theory of the Yule process with immigration to its discrete-time counterpart, consider a population of size $n_1+n_2+\cdots +n_p$, sampled from a single run of a CRP. da Silva et al. (2023) study the pairwise correlation and sample variance of $S_1^*,S_2^*, \cdots ,S_p^*$, the number of types (or species) appearing in the first $n_1$ arrivals, the second $n_2$ arrivals, ..., and the last $n_p$ arrivals of the CRP sample. It is now straightforward from (22) that $(S_1^*,S_2^*, \cdots ,S_p^*)$ is in distribution the same as $(S(t_0,t_1),\cdots ,S(t_{p-1},t_p))$, conditional on observing, in the latter, exactly $n_i$ individuals in $(t_{i-1},t_i)$, for $i=1,\cdots ,p$. As mentioned in the discussion after (17), letting $t_i=\log ((\theta +l_i)/\theta )$ for $i=1,\cdots ,p$, maximizes the chance of observing $n_i$ new individuals (i.e. $n_i$ births) in $(t_{i-1},t_i)$, and under this assumption, the asymptotic behavior of the sample variance of $\tilde{S}_1,\tilde{S}_2,\cdots ,\tilde{S}_p$ and that of the sample variance of $S_1^*,S_2^*, \cdots ,S_p^*$ coincide. In this case, in addition to providing a relatively simpler way to calculate things, the Yule process with immigration allows the sequential samples $\tilde{S}_1,\cdots ,\tilde{S}_p$ to have a random number of individuals, and hence is more appropriate for population models with random sample sizes in which the individuals arrive one by one at random times.

## Discussion

Models for the distribution of the counts $(C_1,C_2,\ldots )$ of family sizes at a particular time (sometimes called counts-of-counts data) have been extensively used in the biological sciences, for example in population genetics, ecology, sampling theory and cancer research. In the present paper, we explored properties of a model arising as the family size counts of samples taken sequentially from a population whose evolution is governed by a birth process with immigration. More specifically, using the theory of marked Poisson processes, we studied the expected sample variance of $S(t_0,t_1),\dots , S(t_{p-1},t_p)$, the families observed in disjoint consecutive time intervals $(t_0,t_1),\dots , (t_{p-1},t_p)$. We explored asymptotic behaviors of the expected sample variance for two types of time intervals, those with equal size and those with logarithmically equal size. The latter was applied to address the Fisher’s sample variance problem for the BI process.

In his 1943 paper, Fisher studied the sample variance of the number of species found in *p* correlated samples. Fisher’s model assumes that the number of species observed *j* times in a typical sample is Poisson distributed with mean $\theta x^j/j$ for $\theta >0, x\in (0,1]$. Under this assumption, one can easily see that the total number of species observed in a sample is a Poisson random variable with mean $-\theta \log (1-x)$, while the total number of specimens in the sample is a negative binomial random variable where the chance of observing exactly *n* specimens in a sample is given by

$$\begin{aligned} \left( {\begin{array}{c}\theta +n-1\\ n\end{array}}\right) (1 - x)^\theta x^n, n=0,1,2 \ldots \end{aligned}$$

From (21), this also implies that, conditional on observing *n* specimens in a sample, the distribution of the counts of species sizes is given by ESF $\mathcal {E}_n$ in (6). Albeit that the correlation of the samples is not clearly described in Fisher’s model, the expected sample variance he derives is asymptotically $\theta \log 2$, which is in contrast with the expected value $\approx \theta \log n$ of the variance of the number of species observed in a sample of size *n* obtained from $\mathcal {E}_n$. Fisher’s result was the subject of various studies including Anscombe (1950) and Watterson (1974b). Conditional on the samples sizes, da Silva et al. (2023) explain Fisher’s results in a discrete-time sequential sampling setting. da Silva et al. (2023) explained Fisher’s multisampling problem using a collection of disjoint samples drawn sequentially from a single run of a CRP. They studied the correlation and sample variance of $S^*_1, S^*_2,\dots ,S^*_p$, the number of species appearing in the first $n_1$ arrivals, the second $n_2$ arrivals, ..., and the last $n_p$ arrivals of a single run of a CRP. They investigated the asymptotic behavior of the variance under different regimes and revisited the Fisher’s log-series distribution as the limiting distribution of the number of types only observed in one particular sample, as the sample sizes proportionally grow.

Despite providing a sequential realization of Fisher’s model and in particular obtaining his variance formula $\theta \log 2$ asymptotically, da Silva et al. (2023) assumes the samples arrive at discrete times and are of fixed sizes $n_1,n_2,\dots$. The latter assumption is indeed equivalent to conditioning on the sample sizes $n_1,n_2,\dots$. Using a random embedding of the CRP into the BI process, in this paper we relaxed these two restrictions and studied the correlation and expected sample variance of $S(t_0,t_1),\dots , S(t_{p-1},t_p)$ obtained from a single run of the BI process. Not only does this allow for samples of random sizes whose size distribution coincides with that of sample sizes in Fisher’s model, but it also provides a framework in which samples arrive continuously in real random times. On the other hand, the embedding of the families into the BI process by recording the actual arrival time of each individual is computationally useful, as it makes families evolve independently. This allows us to apply the theory of the marked Poisson processes in this paper, making the calculations much easier for the BI process. In Sect. 6, we provided the full details of the embedding theory and its connections with well-known conditioning relations. We showed that $(S^*_1,\dots ,S^*_p)$ is in distribution the same as $(S(t_0,t_1),\dots , S(t_{p-1},t_p))$ conditional on having $n_i$ individuals in $(t_{i-1},t_i)$ for $i=1,\dots ,p$.

The correlation and expected sample variance for Fisher’s problem discussed above is related to a very specific case in which the sample interval lengths are logarithmically equal, that is $t_i=\log (i\gamma +1)$, for $i\in \mathbb {Z}_+$ and fixed $\gamma >0$. In this paper, we studied the correlation and expected sample variance of $(S(t_0,t_1),\dots , S(t_{p-1},t_p))$, for a very general choice of intervals in which $0=t_0<t_1<\dots <t_p$. In particular, in Sect. 4.4 we investigated the effects of the parameters $\theta$ and $\lambda$ and interval lengths on the expected sample variances $\mathcal {V}$ and $\mathcal {V}'$ for two types of intervals, the intervals with equal sizes and the ones with logarithmically equal sizes. For instance, Table 1 compares the simulated and exact values for both types of intervals. For fixed $\theta >0$, we observed that as $\lambda$ tends to infinity, $\mathcal {V}\nearrow \theta \tau (\theta \tau +2)(p+1)/(12p)$ while $\mathcal {V}'\searrow 0$ (cf. Proposition 1). On the other hand, for fixed $\lambda >0$, we have $\mathcal {V},\mathcal {V}'\nearrow \infty$ as $\theta \rightarrow \infty$ but the increase is more significant for $\mathcal {V}$ as $\mathcal {V}=O(\theta ^2)$ and $\mathcal {V}'=O(\theta )$.

The theory developed in this paper can be used to infer the parameters $\theta$ and $\lambda$. For $\lambda =1$, one estimator of $\theta$ is given in (15). Another useful example can be given by solving the equations

23 $$\begin{aligned} \hat{S}_i= & {} \frac{\theta }{\lambda }\log (e^{\lambda t_i}-e^{\lambda t_{i-1}}+1), \ \ i=1,\dots ,p\nonumber \\ \hat{N}_i= & {} \frac{\theta }{\lambda }(e^{\lambda t_i}-e^{\lambda t_{i-1}}), \ \ i=1,\dots ,p, \end{aligned}$$

for $\theta$ and $\lambda$, where the left side of the equations stand for the number of families and the number of individuals observed in $(t_{i-1},t_i)$ in the data and the right hand sides are the expected values $\mathbb {E}S(t_{i-1},t_i)$ and $\mathbb {E}Z(t_i)-\mathbb {E}Z(t_{i-1})$. Each time interval $(t_{i-1},t_i)$, for $i=1,\dots ,p$, corresponds to an observer *i* who counts the numbers $\hat{S}_i$ and $\hat{N}_i$ from the data. The *i*-th observer then infer the values of $\theta$ and $\lambda$ by the solutions $\hat{\theta }_i$ and $\hat{\lambda }_i$ of the *i*-th equations in (23). In other words, we obtain *p* inferences of $\theta$ and $\lambda$, namely $\hat{\theta }_i$ and $\hat{\lambda }_i$ for $i=1,\dots ,p$, corresponding to intervals $(t_0,t_1),\dots , (t_{p-1},t_p)$. As a result, the means $\bar{\theta }$ and $\bar{\lambda }$ of $\hat{\theta }_1,\dots , \hat{\theta }_p$ and $\hat{\lambda }_1,\dots ,\hat{\lambda }_p$ can be considered as estimators of $\theta$ and $\lambda$. In this case, the sample variances of $\hat{\theta }_1,\dots , \hat{\theta }_p$ and $\hat{\lambda }_1,\dots ,\hat{\lambda }_p$ measures the variabilities of these estimators and may be regarded as the estimation errors for $\bar{\theta }$ and $\bar{\lambda }$. On the other hand, the expected sample variance of $S(t_0,t_1),\dots , S(t_{p-1},t_p)$ measures the variability of the number of families observed in these intervals and shows how this expected values depends on the interval sizes, as discussed in Sect. 4.4.

## Data Availability

Data sharing not applicable to this article as no datasets were generated or analyzed during the current study.
